# Ohio State Dental Society

**Published:** 1865-11

**Authors:** 


### OHIO STATE DENTAL SOCIETY.

The organization of a State Dental Society, would, we think, meet with the hearty concurrence of the Profession throughout the State, and be the means of great good. It would not in the least operate against any of the local societies now in the State, but would probably be advantageous to them all. Into a State

Society, many members of the profession would be brought who are now out of the reach of the present societies; and it would have a tendency to unite the profession of the whole State in one common cause, which would certainly be productive of great good. We hope that such a move will soon be made, and that a call will soon be issued; and that the profession will be brought together in large numbers, and organize such a society, as will be eminently efficient. It can be done. We hope such a call will be made at an early day.

